# Supplementary material for: Altermagnetic Band Splitting in 10 nm Epitaxial CrSb Thin Films
Source: Adv Mater. 2025 Sep 6;37(47):e08977. doi: 10.1002/adma.202508977 (PMC12651131; doi:10.1002/adma.202508977)
Supplement: Supplementary file 1 — Supporting Information [file ADMA-37-e08977-s001.pdf]

ADVANCED MATERIALS

Supporting Information

for *Adv. Mater.*, DOI 10.1002/adma.202508977

Altermagnetic Band Splitting in 10 nm Epitaxial CrSb Thin Films

Sandra Santhosh, Paul Corbae, Wilson J. Yánez-Parreño, Supriya Ghosh, Christopher J. Jensen, Alexei V. Fedorov, Makoto Hashimoto, Donghui Lu, Julie A. Borchers, Alexander J. Grutter, Timothy R. Charlton, Saurav Islam, Diana Golovanova, Yufei Zhao, Aria Tauraso, Anthony Richardella, Binghai Yan, K. Andre Mkhoyan, Christopher J. Palmstrøm, Yongxi Ou and Nitin Samarth**

Supplementary Material: Altermagnetic band splitting in 10 nm epitaxial CrSb thin films

Sandra Santhosh[1], Paul Corbae[2,3], Wilson J. Yánez-Parreño[2,3], Supriya Ghosh[4], Christopher J. Jensen[5], Alexei V. Fedorov[6], Makoto Hashimoto[7], Donghui Lu[7], Julie A. Borchers[5], Alexander J. Grutter[5], Timothy R. Charlton[8], Saurav Islam[1,9], Diana Golovanova[10], Yufei Zhao[10], Aria Tauraso[1], Anthony Richardella[1,9], Binghai Yan[1,10], K. Andre Mkhoyan[4], Christopher J. Palmstrøm[2,3,11], Yongxi Ou[1*], Nitin Samarth[1,9,12*]

[1]Dept. of Physics, Pennsylvania State University, University Park, 16802, PA, USA.
[2]Dept. of Electrical and Computer Engineering, University of California, Santa Barbara, 93106, CA, USA.
[3]Quantum Foundry, University of California, Santa Barbara, 93106, CA, USA.
[4]Dept. of Chemical Engineering and Materials Science, University of Minnesota, Minneapolis, 55455, MN, USA.
[5]National Institute of Standards and Technology, Gaithersburg, 20899, MD, USA.
[6]Advanced Light Source, Lawrence Berkeley National Laboratory, Berkeley, 94702, CA, USA.
[7]Stanford Synchrotron Radiation Lightsource, SLAC National Accelerator Laboratory, Menlo Park, 94025, CA, USA.
[8]Oakridge National Laboratory, Oak Ridge, 37830, TN, USA.
[9]Materials Research Institute, Pennsylvania State University, University Park, 16802, PA, USA.
[10]Department of Condensed Matter Physics, Weizmann Institute of Science, Rehovot, 7610001, Israel.
[11]Dept. of Materials Science and Engineering, University of California, Santa Barbara, 93106, CA, USA.
[12]Dept. of Materials Science and Engineering, Pennsylvania State University, University Park, 16802, PA, USA.

*Corresponding author(s). E-mail(s): yongxiou@hotmail.com;
nxs16@psu.edu;

1 Influence of substrate temperature on CrSb growth

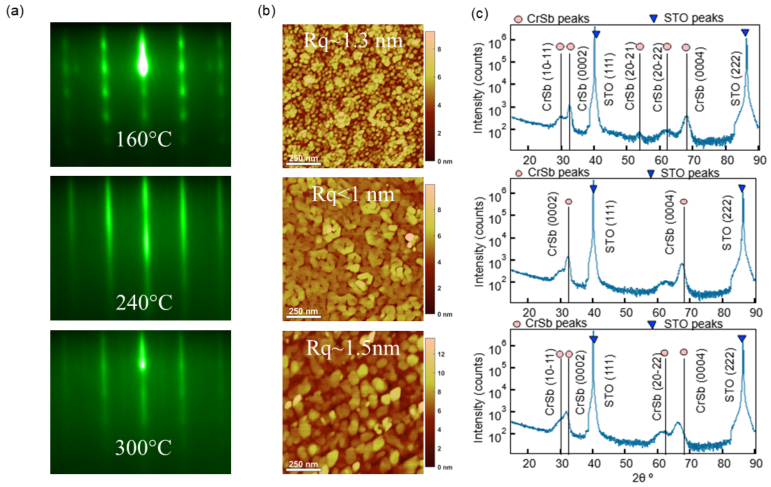

Fig. S 1 Temperature-dependent MBE growth of the Sb_2Te_3(2nm)/CrSb(10nm) films (top: 160°C, middle: 240°C, bottom: 300°C) at a fixed Cr-to-Sb flux ratio of 1:3.75. a) RHEED patterns, **b)** AFM topography images, and **c)** XRD $2\theta - \omega$ scan of Sb_2Te_3/CrSb heterostructures.

Here, we discuss how the deposition conditions during molecular beam epitaxy (MBE) affect the structural quality of CrSb films of thickness 10 nm. We first focus on the role of the substrate temperature. Fig. S1 shows the reflection high energy electron diffraction (RHEED), atomic force microscopy (AFM), and X-ray diffraction (XRD) characterization for a set of CrSb samples grown at three different substrate temperatures: T_S = 160°C (sample A), T_S = 240°C (sample B), and T_S = 300°C (sample C). All three films have the same nominal thickness for each layer, $SrTiO_3$/Sb_2Te_3(2nm)/CrSb(10nm), and are deposited using an identical Cr-to-Sb BEPR of 1:3.75. The RHEED patterns (Fig. S1 (a)) and AFM scans (Fig. S1 (b)) indicate that the epitaxy and surface topography are strongly affected by the growth temperature. The XRD scans with labeled reference peaks [1] indicates crystalline quality of the films and the shift in out of plane lattice constants with respect to the growth temperature.

For sample A deposited at 160°C, RHEED presents relatively faint lines with superposed visible spots, indicating polycrystalline growth due to a low substrate temperature. AFM data is consistent with this picture: Fig. S1(b)(top) shows grain

sizes smaller than 100 nm, with most the grains even smaller than 50 nm. The AFM scan indicates surface roughness with Rq $\sim$ 1.3 nm. Fig. S1(c)(top) shows the XRD $2\theta - \omega$ scan of the sample, showing that, in addition to the (0002) and (0004) CrSb peaks, the (10-11),(20-22) and (20-21) CrSb peaks are also present. This is again consistent with the polycrystalline growth resulting from a low substrate temperature.

Fig. S1 middle panel (sample B) shows the RHEED, AFM, XRD characterization for a 10 nm CrSb sample grown at a substrate temperature of $T_S = 240°$C and a Cr-to-Sb BEPR of 1:3.75. The RHEED pattern (Fig. S1 (a)is similar to Fig 1(b) discussed earlier, consistent with the near-optimal condition for layer-by-layer deposition in the Frank-van der Merwe growth mode. AFM scans of the sample, shown in Fig. S1(b), indicate large grains with typical lateral sizes of about 100 nm or more, accompanied by a small surface roughness (Rq less than 1 nm). Fig. S1(c) indicates that misoriented (10-11) and (20-22) CrSb grains are minimized compared other growth conditions (see also Fig. S2). We note that the XRD spectrum for a thicker sample (31 nm, Fig.1(c)) grown at the same temperature does not show (10-11) and (20-22) peaks. This suggests that misoriented growth occurs near the buffer layer and may anneal out as the film grows thicker and the CrSb (0001) planes become dominant. We also note that the structure factors of the (10-11) to (002) peak are 1000:52 [2] and the XRD plots are on a log intensity scale, implying a only small fraction of the grains are misoriented.

We also investigate the CrSb growth mode at an elevated substrate temperature in sample C ($T_S = 300°$C). The RHEED pattern for this sample consists of streaks with fainter spots than sample A, indicating the presence of certain regions of three-dimensional (3D) island growth along with regions of layer-to-layer epitaxial growth. The combination of 3D island and epitaxial growth modes at this temperature is seen in the AFM scan shown in Fig. S1(b)(bottom). While the average grain sizes of this sample is comparable to sample B, the surface roughness is larger (around 1.5 nm). In Fig. S1(c)(bottom), the XRD $2\theta - \omega$ scan is dominated by the (0002) and (0004) peaks, with a larger shift in the out-of-plane lattice constant from the reference. The comparatively larger counts of (10-11) peak suggest that at this elevated growth temperature, the Sb_2Te_3 buffer layer may start to desorb, degrading its utility in helping nucleate an oriented CrSb film. There are still large regions of epitaxial buffer layer, resulting in the layer-by-layer growth of CrSb leading to the dominant (0002) and (0004) peaks. This elevated growth temperature appears to enable oriented growth within each CrSb grain at the cost of sacrificing a smooth layer-by-layer growth of the sample, resulting in a poor surface roughness.

2 Influence of Cr-to-Sb flux ratio on CrSb growth

Next, we investigate how the Cr-to-Sb beam flux ratio influences the MBE growth of CrSb thin films. For this, we deposit samples with the identical heterostructure, $SrTiO_3/Sb_2Te_3$(2nm)/CrSb(10nm). For this set of samples, we maintain the substrate temperature $T_S = 240°$C but vary the Cr-to-Sb flux ratio during the MBE deposition. The RHEED patterns, AFM topography, and the XRD $2\theta-\omega$ spectra are summarized in Fig. S2. As shown below, the CrSb thin-film crystallinity and surface topography are very sensitive to the Cr-to-Sb flux ratio.

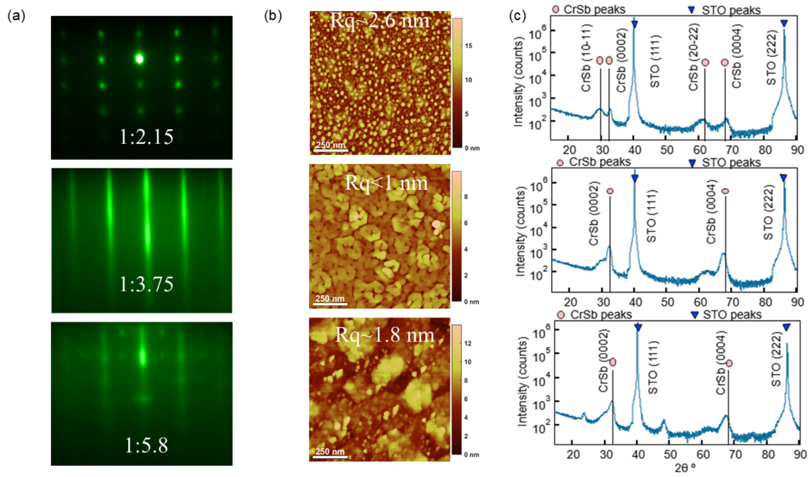

Fig. S 2 Cr-to-Sb flux ratio influence on the MBE growth of the Sb_2Te_3/CrSb(10nm) films (top: 1:2.15, middle: 1:3.75, bottom: 1:5.8) at a fixed temperature of 240°C. a) RHEED patterns, **b)** AFM topography images, and **c)** XRD $2\theta - \omega$ scan of Sb_2Te_3/CrSb heterostructures.

The RHEED pattern for the sample grown using a flux ratio of 1:2.15 (sample D) is shown in Fig. S2(a)(top). Clear spots located on very faint lines are visible in that RHEED image. The spotty RHEED patterns suggest that 3D islands are forming on the sample surface. The AFM scan of the sample is shown in Fig. S2(b)(top), where most grains show sizes of around 50 nm or smaller. The surface roughness of this sample is around 2.6 nm. In the XRD $2\theta - \omega$ scan, the oriented (0002) and the (0004) CrSb peaks, as well as the misoriented (10-11) peaks, are all present, as shown in Fig. S2(c)(top). The (10-11) and (20-22) relative peak intensities also appear to be larger than samples A, B and C, suggesting a worse epitaxy in the film.

Fig. S2(middle panel) repeats the results for sample B grown with a BEPR of 1:3.75 shown in Fig. S1. We note that by increasing the Sb flux from 1:2.15 to 1:3.75, the epitaxy of the thin film significantly improves, as indicated by the streaky RHEED lines in Fig. S2(a)(middle). The AFM scan in Fig. S2(b)(middle) indicates the grain sizes of this sample become larger with increased Sb flux. The XRD scan also supports a better epitaxy in the film, as evident in Fig. S2(c)(middle), where the oriented (0002) and (0004) peaks are enhanced compared to sample E.

As we further increase the Sb flux to a BEPR of 1:5.8 (sample E), the thin film epitaxy becomes worse. The RHEED image of the sample using this flux ratio is shown in Fig. S2(a)(bottom), which forms lines with spots. There is coexistence of small and large grains with sizes varying from a few tens to hundreds of nm as shown in the AFM topography in Fig. S2(b)(bottom). Similar to sample D, the (10-11) and (20-22) CrSb peaks in the XRD $2\theta - \omega$ scan in Fig. S2(c)(bottom) show that the misoriented grains are enhanced and new unidentified peaks begin to appear. Here, we note that the optimized BEPR for the MBE growth of CrSb appears to also depend on the

growth rate of the material, as evident from the comparison between sample in Fig. 1 and sample B. In the parameter space of flux ratio versus growth temperature we identify that the ideal growth conditions occur at 240°C and 1:3.75 flux ratio at a growth rate of 0.04 nm/min. Under this growth rate and growth temperature, the flux ratio of 1:5.8 gives poor CrSb film quality. However, for the thicker sample depositing with a higher growth rate of 0.2 nm/min, it shows that the flux ratio of 1:5.8 appears more preferable when the same growth temperature of 240°C is used.

3 Surface reconstruction in RHEED patterns

We also investigate how the surface reconstruction of the CrSb thin films is affected by the Cr-to-Sb flux ratio. Fig. S3 shows the RHEED surface reconstructions along the 0° and 30° azimuths for samples growing with different Cr-to-Sb flux ratios under the same growth temperature of 240°C. Fig. S3(a) shows the RHEED images of a sample with a CrSb thickness of 10 nm using the same growth conditions as a good epitaxial sample. With a BEPR of 1:4.30, the CrSb film RHEED pattern develops into an apparent (1×4) reconstruction with streaky lines when the film thickness reaches 10 nm. We note that these clear reconstruction lines persist even for thicker films under the same growth conditions. When Sb flux is reduced, the reconstruction changes in a manner that depends on the film thickness. Fig. S3(b) shows the RHEED patterns of a CrSb thin film using a lower Sb flux ratio with BEPR of 1:3.06. Under this condition, the thin film first develops into a (2×2) surface reconstruction when the thickness reaches around 5 nm. As the growth continues, the RHEED pattern becomes spotty, suggesting 3D island growth. These varied surface reconstructions again highlight the sensitivity of the CrSb growth to the Cr-to-Sb flux ratio. These observations are consistent with the previous study on surface reconstructions on MBE-grown CrSb (0001) thin films [3]. Here, we also compare the ARPES band spectrum along $\bar{K}-\bar{\Gamma}-\bar{K}$ in the 10 nm film with (1×4) and the 5 nm film with (2×2) surface reconstructions. Fig. S3(a) bottom and Fig. S3(b) bottom images show that the surface reconstructions hardly modify the low energy bands near the fermi surface.

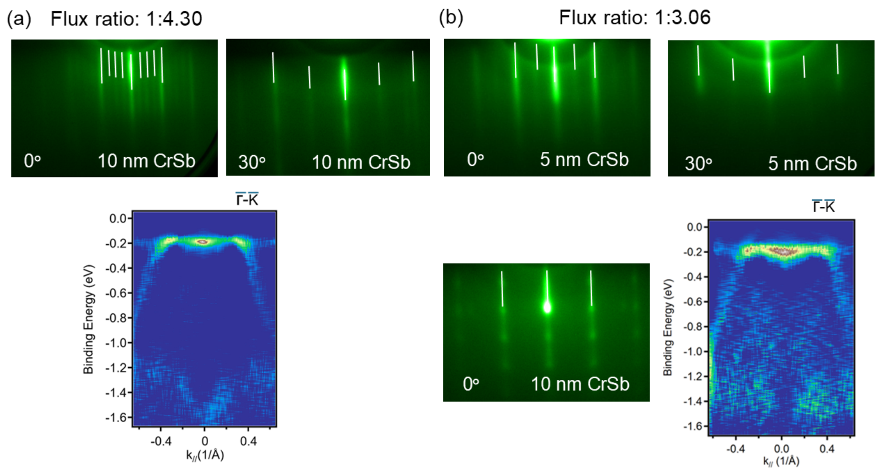

Fig. S 3 : RHEED images showing the varied surface reconstructions for CrSb thin films. RHEED along two azimuths using a Cr-to-Sb flux ratio of **a)** 1:4.3 and **b)** 1:3.06, comparing the low spectral intensity ARPES bands near the fermi energy.

4 X-ray photoemission spectroscopy measurements

To quantify the composition of our samples, we performed X-ray photoemission spectroscopy (XPS) measurements in the CrSb films after angle resolved photoemission spectroscopy measurements (ARPES). The film was kept under ultra-high vacuum (UHV) with a pressure lower than 9×10^{-10} torr at all times before the measurement. We performed a full range scan (Fig. S4 (a)) and see the presence of the expected Cr (3s, 3p, 2p and auger) and Sb (4s, 4p, 3d, 3p and auger) peaks. We also see the presence of small traces of C in the film (C 1 s peak) probably associated with normal outgassing during UHV transfer of the sample. We do not see the presence of oxygen in this sample. We quantified the composition of the film by integrating the area under the Cr $2p_{3/2}$ and Sb $3d_{5/2}$ peaks with a linear background (see Fig. S4 (b) and (c)). We then proceed to normalize these values with the Scoffield sensitivity factors (7.78 for Cr $2p_{3/2}$ and 16.39 for Sb 3d5/2) and obtained the film to be 41% Cr and 59% Sb. This is probably a signature that the surface of the film corresponds to the Sb termination.

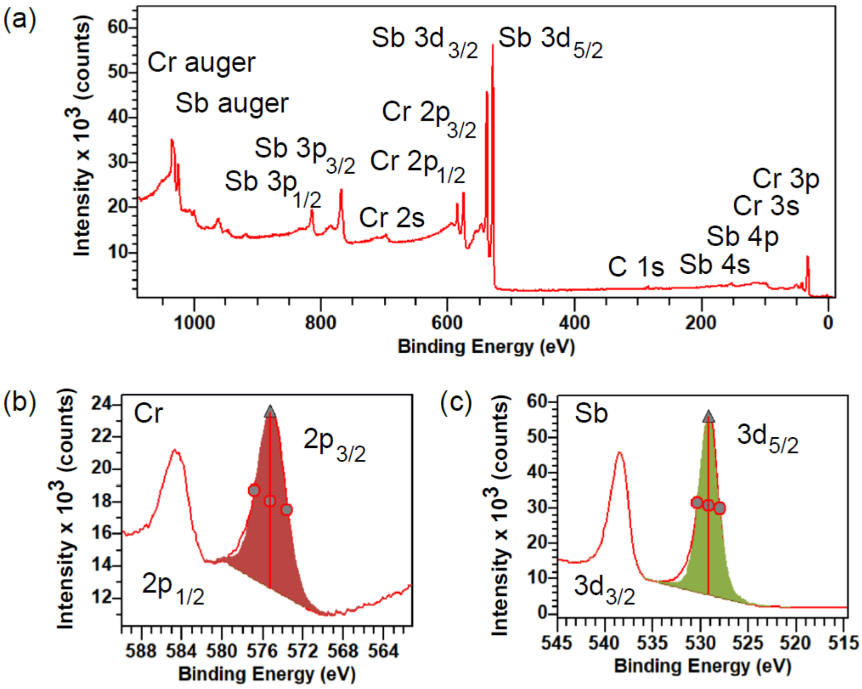

Fig. S 4 : X-ray photoemission spectroscopy (XPS) data of CrSb taken after angle resolved photoemission spectroscopy (ARPES) measurements. a) Full range scan showing the presence of Cr (3s, 3p, 2p and auger) and Sb (4s, 4p, 3d, 3p and auger) and C (1s) peaks in the film. **b)** Small range scan showing the Cr $2p_{3/2}$ and $2p_{1/2}$ peaks. We integrated the area under the Cr $2p_{3/2}$ peak with a linear background to quantify the film composition. **c)** Small range scan showing the Sb $3d_{3/2}$ and $3d_{5/2}$ peaks. We integrated the area under the Sb $3d_{5/2}$ peak with a linear background to quantify the film composition.

5 Electrical transport measurements of a 10 nm thick CrSb epitaxial film

The electrical properties of a $SrTiO_3/Sb_2Te_3$(2nm)/CrSb(10nm) sample are measured using a mechanically defined Hall bar of dimensions 1 mm × 0.5 mm. Fig. S5(a) shows the sample's longitudinal resistance (R_{xx}) versus temperature and Fig. S5(b) shows the the Hall resistance (R_{xy}) as a function of the out-of-plane magnetic field at different temperatures. All measurements are performed with an excitation current of 100 μA. We estimate a room-temperature longitudinal resistivity of 50 Ωcm. The temperature-dependent R_{xx} shows a typical metallic behavior, with a residual resistivity ratio ≈ 2. Below T = 12 K, we observe an upturn in resistance, possibly due to electron-electron interactions. The hysteresis in the anomalous Hall effect (AHE) persists till T = 20 K, while the non-linear AHE itself survives till 150 K. The Hall resistance is linear above T = 200 K. For the g-wave altermagnet, CrSb, without additional broken

symmetry, the AHE is forbidden [4]. In view of the PNR results showing evidence of ferromagnetism in the thin Sb_2Te_3 buffer layer, we attribute the AHE to the diffusion of Cr into this buffer. This can readily create a trivial conducting ferromagnet.

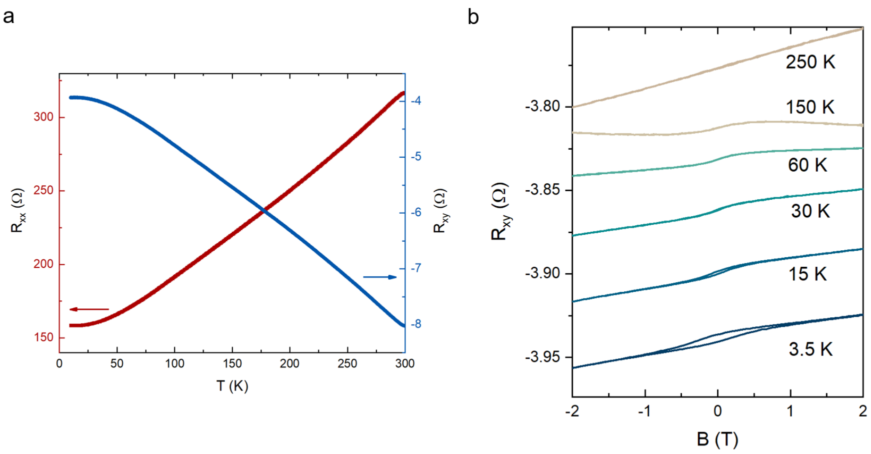

Fig. S 5 : Electrical transport measurements on CrSb a) The longitudinal resistance (R_{xx}) in a 10 nm thick CrSb film as a function of temperature (T) **b)** Transverse resistance (R_{xy}) at different temperatures in the same sample, showing hysteresis and AHE.

6 HAADF-STEM and EDX spectroscopy on CrSb

Figure S6(a) shows a high angle annular dark field scanning transmission electron microscopy (HAADF-STEM) image of the CrSb (31 nm)-Sb_2Te_3-$SrTiO_3$ heterostructure, showing epitaxial growth of the CrSb film over the buffer layer. The elemental distribution and composition in these MBE-grown CrSb thin films were evaluated using STEM energy-dispersive X-ray (EDX) spectroscopy elemental mapping (Fig. S6(b)). Using compositional line-scans from the EDX maps shown in Fig. S6 (c), we determined the Cr to Sb ratio to be 1:1. The samples were not capped prior to the measurements, the 10 at% oxygen signal seen in the CrSb layer is likely due to oxygen diffusion during the TEM sample preparation.

We now make a few comments about the nature of the buffer layer (intended to be Sb_2Te_3). STEM-EDX data from multiple regions of the film show areas where the Sb_2Te_3 layer is present, but with Cr also detected in these regions. It is possible that Cr diffuses into the van der Waals gap of the Sb_2Te_3 layer. Another possibility is the formation of $CrTe_2$ in some regions of the film. This may be consistent with the absence of an obvious van der Waals gap in some regions of the buffer. We note that there are a few regions of the sample where a buffer layer seems to be absent. However, the film is (0001) out of plane oriented in all areas examined, regardless of the presence of a buffer or not.

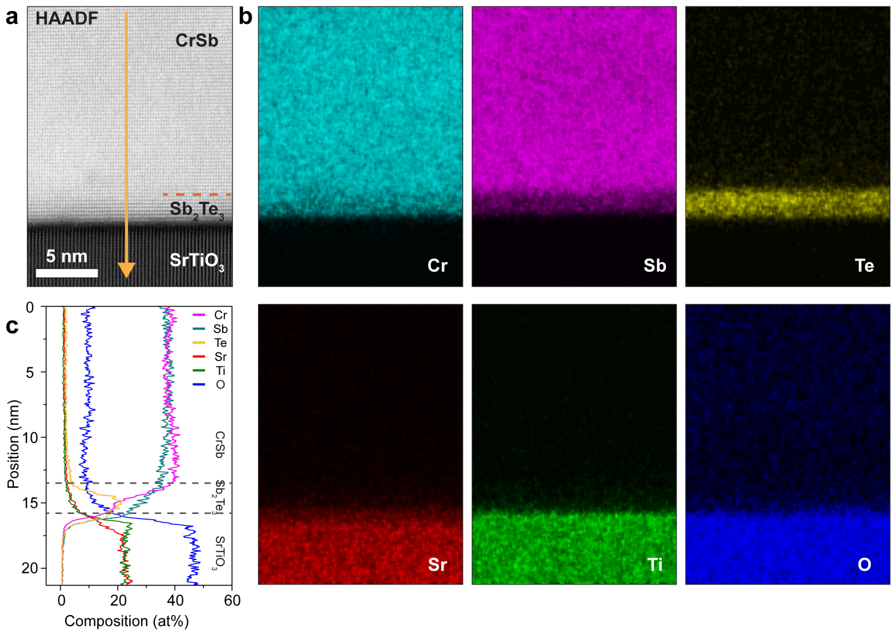

Fig. S 6 : HAADF-STEM and EDX spectroscopy on CrSb. a) HAADF-STEM image of the CrSb-Sb_2Te_3-$SrTiO_3$ heterostructure with the different layers indicated. **b)** STEM-EDX elemental maps from the region in (a) for Cr, Sb, Te, Sr, Ti and O. **c)** Line-scan of the EDX signal obtained from the region in the panel (a) showing the relative composition of the elements present in the heterostructure.

7 Polarized Neutron Reflectivity

Chosen Model Reflectivity and Spin Asymmetry

The polarized neutron reflectivity (PNR) fits for the Te (10 nm)/CrSb (x nm)/Sb_2Te_3 (2 nm)/$SrTiO_3$ (111) are shown below for the x = 100 nm (Fig. S7(a) and x = 50 nm samples (Fig. S8(b). Spin asymmetry [(R+ - R-) / (R+ + R-)] was calculated to highlight the difference between R+ and R-, which arises from the presence of magnetic moments in the structure. This is shown for the 100 nm CrSb sample (Fig. S7(b) and 50 nm CrSb sample (Fig. S8(c) below. Fig. 1(f) (main text) shows the reconstructed nuclear (ρ) and magnetic (ρ_M) scattering length density profiles for the 100 nm CrSb sample, and Fig. S8(a) shows the same for the 50 nm CrSb sample. The results are presented and discussed in the main text of this paper.

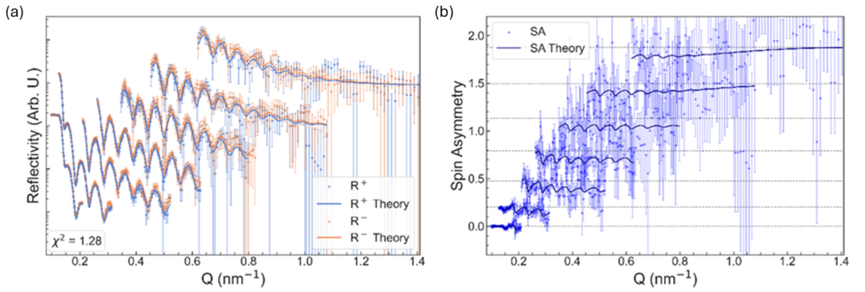

Fig. S 7 : a) Polarized neutron reflectivity data (points) and theoretical fits (lines) for the chosen model of the Te (10 nm)/CrSb(100 nm)/Sb_2Te_3 (2 nm)/$SrTiO_3$ (111) sample, plotted with vertical offsets between the different measurement angles for clarity. **b)** the spin asymmetry, (R+ - R-)/(R+ + R-), for the corresponding reflectivity at each measurement angle, plotted with vertical offsets for clarity and a horizontal dashed line that represents spin asymmetry = 0. Error bars represent ± 1 std. deviation.

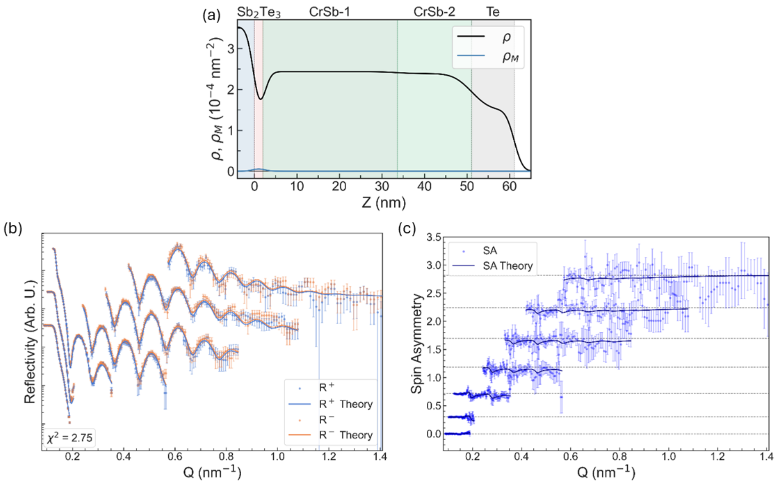

Fig. S 8 : a) PNR ρ and ρ_M profiles calculated from fits using the chosen model of the Te (10 nm)/CrSb(50 nm)/Sb_2Te_3 (2 nm)/$SrTiO_3$ (111) film measured at 300 K in a 1 T magnetic field. Background colors were used to highlight each layer in the model, with the corresponding label above the plot. The blue background (left) corresponds to the $SrTiO_3$ substrates, and the white background (right) corresponds to air. **b)** Polarized neutron reflectivity data (points) and theoretical fits (lines) for the chosen model of the Te (10 nm)/CrSb(50 nm)/Sb_2Te_3 (2 nm)/$SrTiO_3$ (111) sample, plotted with vertical offsets between the different measurement angles for clarity. **c)** the spin asymmetry (R+ - R-)/(R+ + R-) for the corresponding reflectivity at each measurement angle, plotted with vertical offsets for clarity and a horizontal dashed line that represents spin asymmetry = 0. Error bars represent ±1 std. deviation.

Further discussion of PNR fitting process: 50 nm CrSb sample

For the PNR fitting process, a series of models were used to determine a model that matched the known characteristics of the samples' structures without being under or over parameterized. This process was necessary to reduce the number of degenerate models that fit the data equally well (equivalent χ^2), which was performed by starting with the simplest model of the expected structure and increasing complexity until no further improvements were seen in the quality of the fits. The quality of each fit was determined from both the quantitative χ^2 and from the qualitative match with the spin asymmetry (i.e., splitting in R+ and R- due to magnetization in the structure). The latter was important for this system, as the sensitivity of χ^2 to the models' very small magnetization was limited. Additionally, we utilized a Markov Chain Monte Carlo method (Bayesian approach) to perform an uncertainty analysis [5], which allowed us to better understand the significance of the fitted parameter values and to calculate their confidence intervals. Through the series of excluded models shown and discussed below, we demonstrate support for the structural and magnetic profiles of the best fit modes models for the 50 nm film (Fig 1(f) and Fig. S8(a). The models shown here represent a subset of a larger series of models used during the fitting process. Note

that alternate models for the 100 nm film were excluded for similar reasons using the same criteria.

No magnetization Sb_2Te_3 model

This model of the Te (10 nm)/CrSb (50 nm)/Sb_2Te_3 (2 nm)/$SrTiO_3$ (111) sample used similar parameters to the chosen best fit models, except that it did not allow any ρ_M (and thus magnetization) in the Sb_2Te_3 layer. As shown in Fig. S9(a), the CrSb-1 and CrSb-2 sublayers were fit with nearly equivalent values of ρ compared to the best fit model (Fig. S8(a), but with a non-zero ρ_M of 0.0147 $\times$ 10^{-4} nm^{-2} and 0.151 $\times$ 10^{-4} nm^{-2}, respectively, and 95% confidence intervals for ρ_M of 0.0110 - 0.0184 $\times 10^{-4}$ nm^{-2} (CrSb-1) and 0.0108 - 0.0197 $\times$ 10^{-4} nm^{-2} (CrSb-2). The fit has a slightly worse χ^2 compared to the best fit model (2.81 vs. 2.75), and the spin asymmetry (Fig. S9(c) shows that this model clearly does not capture the distribution of the magnetization in the structure correctly. Because of this, we consider this model under parameterized and excluded as a potential solution. Further, this model helps justify the inclusion of a non-zero ρ_M in the Sb_2Te_3 layer to fully capture the spin asymmetry seen in the data.

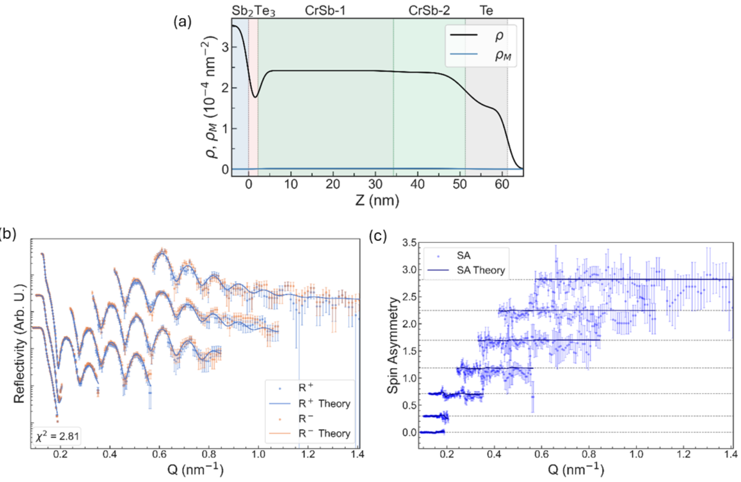

Fig. S 9 : **a)** PNR ρ and ρ_M profiles calculated from fits using the "no magnetization Sb_2Te_3" model of the Te (10 nm)/CrSb(50 nm)/Sb_2Te_3 (2 nm)/$SrTiO_3$ (111) film measured at 300 K in a 1 T magnetic field. Background colors were used to highlight each layer in the model, with the corresponding label above the plot. The blue background (left) corresponds to the $SrTiO_3$ substrates, and the while background (right) corresponds to air. **b)** Polarized neutron reflectivity data (points) and theoretical fits (lines) for the "no magnetization Sb_2Te_3" model of the Te (10 nm)/CrSb(50 nm)/Sb_2Te_3 (2 nm)/$SrTiO_3$ (111) sample, plotted with vertical offsets between the different measurement angles for clarity. **c)** the spin asymmetry (R+ - R-)/(R+ + R-) for the corresponding reflectivity at each measurement angle, plotted with vertical offsets for clarity and a horizontal dashed line that represents spin asymmetry = 0. Error bars represent $\pm$1 std. deviation.

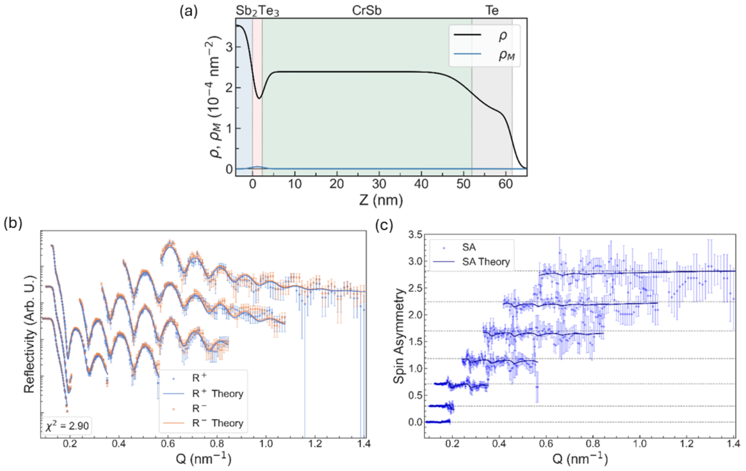

Fig. S 10 : a) PNR ρ and ρ_M profiles calculated from fits using the "single CrSb layer" model of the Te (10 nm)/CrSb(50 nm)/Sb_2Te_3 (2 nm)/$SrTiO_3$ (111) film measured at 300 K in a 1 T magnetic field. Background colors were used to highlight each layer in the model, with the corresponding label above the plot. The blue background (left) corresponds to the $SrTiO_3$ substrates, and the while background (right) corresponds to air. **b)** Polarized neutron reflectivity data (points) and theoretical fits (lines) for the "single CrSb layer" model of the Te (10 nm)/CrSb(50 nm)/Sb_2Te_3 (2 nm)/$SrTiO_3$ (111) sample, plotted with vertical offsets between the different measurement angles for clarity. **c)** the spin asymmetry (R+ - R-)/(R+ + R-) for the corresponding reflectivity at each measurement angle, plotted with vertical offsets for clarity and a horizontal dashed line that represents spin asymmetry = 0. Error bars represent ± 1 std. deviation.

Single CrSb layer model

This model of the Te (10 nm)/CrSb (50 nm)/Sb_2Te_3 (2 nm)/$SrTiO_3$ (111) sample again used similar parameters to the chosen best fit models, except that the CrSb layer was not split into two sublayers, as shown in Fig. S10(a). The CrSb layer ρ was fit to $2.388 \times 10^{-4}\ nm^{-2}$, which falls between the CrSb-1 and CrSb-2 ρ of the best fit model, and ρ_M was similarly negligible. The Sb_2Te_3 magnetization calculated from ρ_M was also similar to the best fit model (30.5 kA m^{-1} vs. best fit 32.6 kA m^{-1}), which also showed good agreement with the spin asymmetry (Fig. S10(c). The reasoning for excluding this model as under parameterized was the increased χ^2 compared to the best fit model (2.90 vs 2.75). This model offers further support for a second CrSb sublayer to improve χ^2.

Inclusion of TeO_x model

The final model shown here of the Te (10 nm)/CrSb (50 nm)/Sb_2Te_3 (2 nm)/$SrTiO_3$ (111) sample is included as an example of where we began to over parameterize our models. This model included all the parameters that were used in the best fit model, except allowed for a secondary "TeO_x" layer to be present at the surface with a varying ρ from the Te layer, as shown in Fig.S11(a). As oxidation may have occurred during the brief exposure to atmosphere while mounting samples, this layer

physically makes sense, yet when including it in the model, minimal improvements are seen. The goodness of fit slightly decreased compared to the best fit model (χ^2 of 2.73 vs. 2.75), and a similar qualitative agreement of the fit to the spin asymmetry is seen. As no meaningful improvements were observed, this model was excluded as a potential solution.

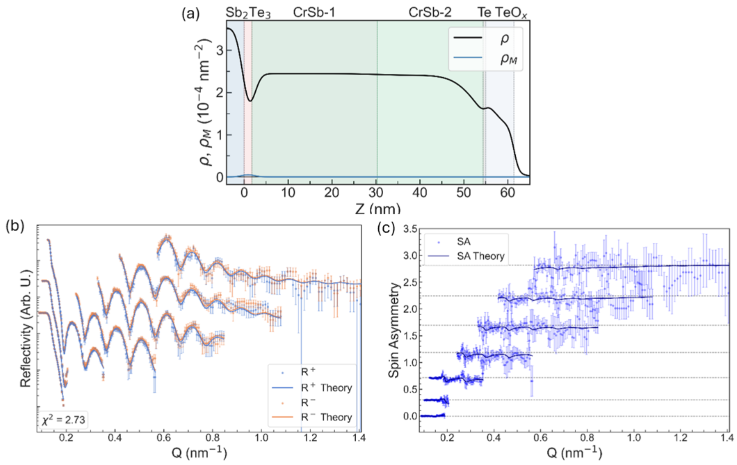

Fig. S 11 : **a)** PNR ρ and ρ_M profiles calculated from fits using the "inclusion of TeO_x" model of the Te (10 nm)/CrSb(50 nm)/Sb_2Te_3 (2 nm)/$SrTiO_3$ (111) film measured at 300 K in a 1 T magnetic field. Background colors were used to highlight each layer in the model, with the corresponding label above the plot. The blue background (left) corresponds to the $SrTiO_3$ substrates, and the while background (right) corresponds to air. **b)** Polarized neutron reflectivity data (points) and theoretical fits (lines) for the "inclusion of TeO_x layer" model of the Te (10 nm)/CrSb(50 nm)/Sb_2Te_3 (2 nm)/$SrTiO_3$ (111) sample, plotted with vertical offsets between the different measurement angles for clarity. **c)** the spin asymmetry (R+ - R-)/(R+ + R-) for the corresponding reflectivity at each measurement angle, plotted with vertical offsets for clarity and a horizontal dashed line that represents spin asymmetry = 0. Error bars represent ±1 std. deviation.

8 X-Ray Reflectivity

Samples were also measured with X-Ray Reflectivity (XRR) finding similar density profiles as in the PNR fits where the CrSb density near the surface becomes lower when the film is beyond about 50 nm thick, as shown in Fig. S12.

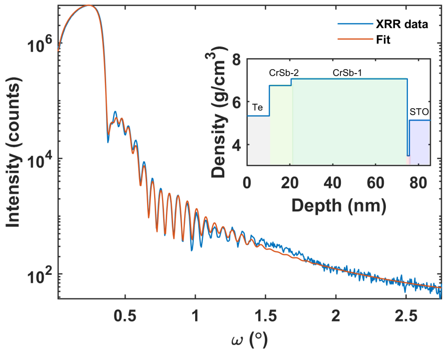

Fig. S 12 : XRR reflectivity curve and fit. Inset shows densities for the layers in the fit (not including smearing of the densities at the interfaces due to the roughness of the layers). Similar to PNR, a decreased density in the CrSb beyond about 50 nm is found to best fit the data.

Material	Thickness (nm)	Density (g/cm^3)	Roughness (nm)
Te	10.45	5.33	3.29
CrSb-2	10.15	6.75	1.28
CrSb-1	54.17	7.06	1.04
Sb_2Te_3	0.97	3.47	1.29
$SrTiO_3$	–	5.13	0.00

Table 1 Parameters from XRR fit

9 Altermagnetic band splitting in 5 nm thin CrSb films

To further investigate the existence of altermagnetic band splitting in samples thinner than 10 nm, we made CrSb (5 nm)/Sb_2Te_3 (2 nm)/$SrTiO_3$ (111) films. The RHEED pattern and the AFM topography of the sample, shown in Fig. S13(a) and (b), confirm the epitaxial nature of the 5 nm thin film with a low surface roughness value of ∼1.2 nm. The ARPES measurements were performed using the He lamp source along the $\bar{M} - \bar{\Gamma} - \bar{M}$ direction to study the presence of band splitting. The $2D$ curvature plot of the sample measured at 300 K in Fig. S13(c) shows band splitting signatures at $k_{||}$ less than zero. The resolution of the splitting is comparatively lower than the 10 nm films possibly due to structural defects in the ultrathin limit, which also makes it difficult to grow samples thinner than 5 nm using our methods. To study the influence of temperature, we also attempted measuring the sample at 77 K (see Fig.S13(d)) but the similar resolution implies that thermal broadening does not modify the bands significantly. It is also evident from the spectrum that even in the ultrathin limit the

bulk bands at and below the binding energy value of -1.5 eV are identical to the 10 nm and 100 nm samples.

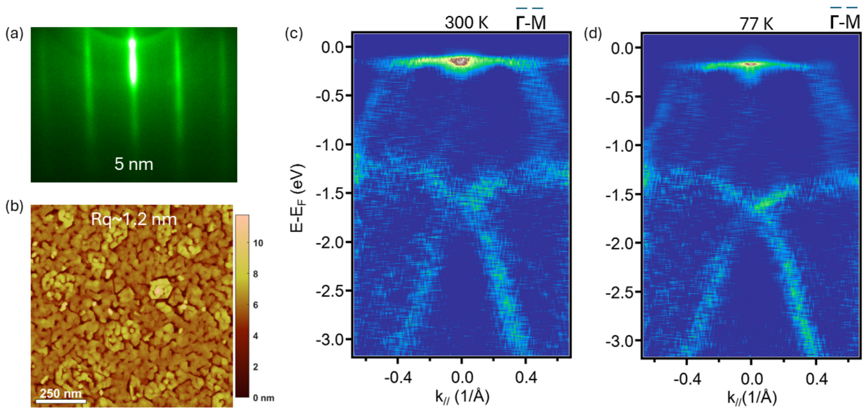

Fig. S 13 : ARPES on 5 nm thin CrSb films. The RHEED pattern **a)** and the AFM topography **b)** of the sample. $2D$ curvature plots of the ARPES band spectrum on 5 nm films indicating band splitting measured at 300 K**c)** and at 77 K **d)**.

10 Band structure calculations of thin CrSb films

To determine the minimum slab thickness required to observe noticeable differences in ARPES measurements, we computed the band structures of CrSb slabs with varying thicknesses. Figure S14(a) shows the bulk band structure along two representative high-symmetry paths: in the middle plane of BZ ($k_z = 0$), the $M - \Gamma - M$ k-path, and on the top of the Brillouin zone ($k_z = 0.5c^*$ where c^* is the reciprocal vector in z direction), the $L - A - L$ k-path. Then we constructed the slabs, leaving the xy-plane periodic and choosing a finite number of unit cells in the z direction.

In the bulk band structure, an electron-like band crosses the Fermi energy at the A point. This band can be pushed to empty states in thin films due to the size effect. For the 1-unit-cell slab (Fig. S14(b)), the band structure deviates significantly from the bulk: the bands are generally pushed to higher energies, and no state is observed near the Fermi level at the Γ point, different from ARPES. Similar deviations are seen in the 2- and 3-unit-cell slabs Figs. S14(c)–(d). At a slab thickness of 4 unit cells (Fig. S14(e)), the band structure at the Γ point begins to resemble the bulk profile along the L–A–L path. As the thickness increases to 5 and 6 unit cells (Figs. S14(f)–(g)), this band progressively lowers in energy and eventually crosses the Fermi level. By 7 unit cells (Fig. S14(h)), the band reaches the same energy as in the bulk, with further confirmation seen for 9- and 18-unit-cell slabs (Fig. S14(i)–(j)). Since ARPES is sensitive to the electronic states near the Γ point, these results suggest that

clear changes near Fermi level should become visible in experiments for a critical slab thicknesses of 5/6 unit-cell thick, about 3 nm.

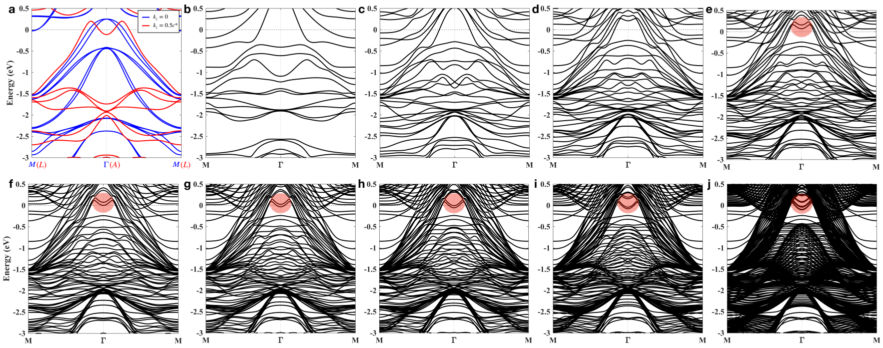

Fig. S 14 : DFT calculations of band structure of altermagnetic CrSb thin films inlcuding spin orbit coupling. (a) Bulk band structure. Blue lines correspond to M-Γ-M ($k_z = 0$) k-path, while red lines correspond to L-A-L ($k_z = 0.5c^*$) kpath. (b) Slab with the width of 1 unit cell in z direction, $l = 5.5$ Å. (c) Slab with the width of 2 unit cells in z direction, $l = 11$ Å. (d) Slab with the width of 3 unit cells in z direction, $l = 16.5$ Å. (e) Slab with the width of 4 unit cells in z direction, $l = 22$ Å. (f) Slab with the width of 5 unit cells in z direction, $l = 27.5$ Å. (g) Slab with the width of 6 unit cells in z direction, $l = 33$ Å. (h) Slab with the width of 7 unit cells in z direction, $l = 38.5$ Å. (i) Slab with the width of 9 unit cells in z direction, $l = 49.5$ Å. (j) Slab with the width of 18 unit cells in z direction, $l = 99$ Å. The red circle on top of the slab's band structures highlights the formation of the band at the Γ point through different widths.

Density-functional theory (DFT) calculations were performed using the Vienna ab initio Simulation Package (VASP) with the projector-augmented wave (PAW) method [6, 7]. The exchange-correlation functional was treated using the generalized gradient approximation (GGA) parametrized by Perdew-Burke-Ernzerhof (PBE) [8]. The kinetic energy cutoff for the plane-wave basis was set to 500 eV. A GGA+U implementation was also used with $U_{eff} = 0.25$ eV for the Cr atom, where $U_{eff} = U - J$. The Brillouin zone integration was carried out using an $8 \times 8 \times 8 \Gamma$-centered regular k-mesh. To calculate the slab band structure, the tight-binding model was constructed using the WANNIER90 package [9]. The Maximally localized Wannier functions (MLWFs) of Cr-3p, 3d, 4s and Sb-5p orbitals were considered. The lattice parameters are $a = b = 4.075$ Å and $c = 5.508$ Å, corresponding to the experimental values.

References

[1] Kjekshus, A., Walseth, K.P.: On the properties of the $Cr_{1+x}Sb$, $Fe_{1+x}Sb$, $Co_{1+x}Sb$, $Ni_{1+x}Sb$, $Pd_{1+x}Sb$, and $Pt_{1+x}Sb$ Phases. Acta Chemica Scandinavica **23**, 2621–2630 (1969) https://doi.org/10.3891/acta.chem.scand.23-2621

[2] Grazhdankina, N., Medvedeva, I.: Influence of high pressure on the magnetic properties of alloys $Mn_{1-x}Cr_xSb$. Phys. Met. Metallogr. **55**(1-3) (1983)

[3] Burrows, C.W., Aldous, J.D., Bell, G.R.: Epitaxial growth and surface reconstruction of CrSb(0001). Results Phys. **12**, 1783–1785 (2019) https://doi.org/10.1016/j.rinp.2019.02.021

[4] Zhou, Z., Cheng, X., Hu, M., Chu, R., Bai, H., Han, L., Liu, J., Pan, F., Song, C.: Manipulation of the altermagnetic order in CrSb via crystal symmetry. Nature **638**(8051), 645–650 (2025) https://doi.org/10.1038/s41586-024-08436-3

[5] Vrugt, J.A., Braak, C.J., Diks, C.G., Robinson, B.A., Hyman, J.M., Higdon, D.: Accelerating markov chain monte carlo simulation by differential evolution with self-adaptive randomized subspace sampling. International journal of nonlinear sciences and numerical simulation **10**(3), 273–290 (2009)

[6] Kresse, G., Furthmüller, J.: Efficient iterative schemes for ab initio total-energy calculations using a plane-wave basis set. Phys. Rev. B **54**, 11169–11186 (1996) https://doi.org/10.1103/PhysRevB.54.11169

[7] Kresse, G., Joubert, D.: From ultrasoft pseudopotentials to the projector augmented-wave method. Phys. Rev. B **59**, 1758–1775 (1999) https://doi.org/10.1103/PhysRevB.59.1758

[8] Blöchl, P.E.: Projector augmented-wave method. Phys. Rev. B **50**, 17953–17979 (1994) https://doi.org/10.1103/PhysRevB.50.17953

[9] Perdew, J.P., Burke, K., Ernzerhof, M.: Generalized gradient approximation made simple. Phys. Rev. Lett. **77**, 3865–3868 (1996) https://doi.org/10.1103/PhysRevLett.77.3865
